# The Utility of Troponin in Predicting Cardiac Dysfunction in Pediatric Patients: A Meta-Analysis

**DOI:** 10.7759/cureus.91391

**Published:** 2025-09-01

**Authors:** Rachna Kadakia, Corneila Muntean, Lauren R Klein, Sarah Eckardt, Cameron E Kalin, Lauren Rothburd, Catherine Caronia, Patricia A Eckardt

**Affiliations:** 1 Pediatric Emergency Medicine, Good Samaritan University Hospital, West Islip, USA; 2 Emergency Medicine, Good Samaritan University Hospital, West Islip, USA; 3 Hospital Administration/Process Improvement, Northwell Health, Huntington, USA; 4 Pediatrics, Cohen Children's Medical Center/Long Island Jewish Medical Center, New Hyde Park, USA; 5 Trauma, Good Samaritan University Hospital, West Islip, USA; 6 Pediatrics, Good Samaritan University Hospital, West Islip, USA; 7 Nursing, Good Samaritan University Hospital, West Islip, USA

**Keywords:** cardiac, chest pain, emergency department, pediatric, troponin

## Abstract

In the management of adults, troponins are routinely used to detect acute coronary syndrome and to predict increased risk of mortality. However, there is limited information available on the utility of troponin for risk assessment in children with chest pain. This study aimed to investigate whether elevated troponin in pediatric patients (0-21 years of age, as per guidelines during the sampling timeframe) presenting with chest pain has a role as a prognostic biomarker for myocardial dysfunction. In a systematic review and meta-analysis, searches were scoped with language restriction to English on PubMed and Cochrane from their inception to 2023. Evidence included studies reporting the association of elevated troponin with myocardial dysfunction. A summary of odds ratios for the association of elevated troponin with myocardial dysfunction was generated. The Quality in Prognosis Studies tool was used to assess methodological quality. Six studies involving 3,144 patients presenting with chest pain were included in the analysis. The risk of myocardial dysfunction was greater in subjects with elevated troponin than in those without (odds ratio, 3.30; 95% confidence interval, 2.19-4.40). Considerable interstudy heterogeneity (I^2^ statistic 87%) was observed and partially explained by variations in study design, troponin subtype, and overall risk of bias. Elevated troponin is associated with myocardial dysfunction in children presenting with chest pain in this sample. Further high-quality multisite studies are needed to inform clinical application of these findings.

## Introduction and background

The prevalence of pediatric chest pain accounts for 0.5-1% of all pediatric emergency department (ED) visits. Although chest pain is a common pediatric emergency room presenting complaint, the majority of these pediatric patients are diagnosed with non-cardiac causes based on history and examination [1-3]. Acute chest pain in children without any prior medical history is rarely cardiac in etiology. Non-emergent cardiac factors related to presenting complaints of chest pain include infection, such as pneumonia, inflammation, such as costochondritis, and valvular conditions, including prolapse [4].

Accordingly, acute myocardial infarction (AMI) is uncommon in the pediatric population. Studies have estimated that cardiac chest pain in children ranges from 1% to 8%, with a recent study suggesting 5.1% to 8.6% across children ranging from <1 to 18 years of age [5,6]. However, a precise prevalence rate cannot be accurately estimated due to the multiple pathophysiologic mechanisms that can lead to pediatric cardiac ischemia [7]. Though acute cardiac ischemia is not a common reason for chest pain in children, it does pose a serious risk requiring timely diagnosis and treatment for the best outcomes, particularly in high-risk children [1]. Clinical guidelines and consensus statements have been proposed to assist clinicians in the accurate diagnosis and treatment of pediatric patients with chest pain [8,9]. Still, the differential diagnosis is confounded by a child's age and developmental milestones. For example, in children less than two years of age, chest pain is often only suspected by observed chest tenderness upon chest palpation. Older children often complain of shortness of breath or palpitations associated with chest pain [10]. These diverse presentations and parents' anxiety related to their child's complaint of chest pain can lead to further testing, including serum troponin markers [1].

Serum troponin is a protein found in the cardiac muscle that is released during myocardial injury. It is therefore used as a marker of myocardial cell injury and death [11]. In the management of adults, serum troponins are routinely used to detect acute coronary syndrome and to predict increased risk of mortality [12]. As such, the measurement of troponin levels to evaluate myocardial injury has increasingly become a part of the standard evaluation of cardiorespiratory symptoms [11,13]. This has resulted in elevated serum troponins being more frequently detected in the ED [1,14]. However, there is limited information available on the diagnostic utility of troponin for risk assessment in children with chest pain [15]. For example, both cardiac troponin I (cTnI) and cardiac troponin T (cTnT) are of diagnostic and clinical value in the identification of myocardial injury in adults. However, in children, cTnI and cTnT concentrations in healthy children and adolescents have been found to differ from those in adults, with some studies reporting slight increases, particularly in neonates [14,16]. Additionally, sex-specific differences have been observed in healthy adolescent subjects [17,18]. Yet, multiple studies have reported serum troponin levels associated with acute cardiac injury in children, prompting cardiac biomarkers to be used in pediatric evaluation for chest pain [19,20].

Therefore, the aim of this investigation was to gather data on studies that examined pediatric patients with elevated troponins; the second aim was to gain a better understanding of whether troponin is a useful tool for detecting myocardial dysfunction. The goal was to analyze the studies currently done on the topic in order to see if there is a general conclusion that may help guide clinical practice when ordering troponins in the pediatric ED.

## Review

Methodology

Study Design

This systematic review and meta-analysis of prognostic observational studies was designed using the most recent methodological guidance [21] and was reported in compliance with the Meta-analysis of Observational Studies in Epidemiology guidelines [22]. This study was reviewed by the institution's Institutional Review Board on August 22, 2022, and acknowledged as an exempt category. In conjunction with a medical librarian, we conducted a search of PubMed and Cochrane to include citations from inception to January 3, 2023, and December 14, 2023, respectively. We reviewed the bibliographies of identified studies and review articles for potentially missed articles. This study was conducted in accordance with the Preferred Reporting Items for Systematic Reviews and Meta-Analyses (PRISMA) guidelines.

Eligibility Criteria

Original research studies that reported an association between elevated troponin and myocardial dysfunction were included in this meta-analysis. These studies consisted of retrospective, prospective, or randomized controlled trials. We reviewed studies that involved patients who were previously healthy and did not have pre-existing cardiac pathology. As our sampling frame included studies from inception investigating the relationship between troponin and cardiac dysfunction in pediatric populations, pediatric patients were identified as being 0-21 years old, reflecting the American Academy of Pediatrics and the Consortium of European Pediatric Societies and Associations' upper limit across this timeframe of 2011-2023 [23-25].

Studies were filtered for those published after 2010 and those that had text in the English language. Abstracts and conference presentations, case reports, case series, editorials, expert opinions, publications with incompletely reported data, and nonhuman studies were excluded. Studies were excluded if they looked at patients with a history of structural or functional heart disease. Studies that examined neonates (children under 28 days of age) or patients over the age of 21 were excluded.

Search Strategy

Searches were completed on PubMed and Cochrane from their inception to January 3, 2023, and December 14, 2023. The search strategy included a comprehensive set of search terms for troponin and myocardial dysfunction. The search words "troponin" AND "pediatric" OR "children" OR "infants" OR "adolescents" were used to retrieve studies. The search was filtered to include only studies published after 2010, accounting for advancements in troponin assays and higher sensitivities [26]. A language restriction to studies published in English was entered as a search parameter.

Study Selection

Two authors (RK and CK) independently screened titles and abstracts for potentially relevant studies. The full texts of eligible studies were extracted and assessed independently and in duplicate against the eligibility criteria. Any discrepancies were adjudicated by the joint agreement of all the authors. The authors also reviewed the reference lists of the included studies for additional potentially relevant studies.

Data Extraction and Management

Data from individual studies were abstracted by another author and checked independently by a senior author. Two authors (SE and PE) independently used standardized spreadsheets to extract data from included studies. Where reported, the following were recorded: study design, study location, sample size, patient ages, troponin type, troponin threshold, and outcomes measured. Associations between elevated troponin and myocardial damage were evaluated.

Patients were evaluated for characteristics that would qualify them for inclusion in the treatment and control groups. The treatment positive group included patients who were positive for cardiac dysfunction with elevated troponin levels. The treatment negative group included patients who were negative for cardiac dysfunction with elevated troponin levels.

For categorization within the control group, the control positive group included patients who were positive for cardiac dysfunction with regular troponin levels. The control negative group included patients who were negative for cardiac dysfunction with non-elevated troponin levels.

Assessment of Methodological Quality and Bias Assessment

Two authors (RK and SE) independently assessed the methodological quality of included studies using the Quality in Prognosis Studies (QUIPS) tool, with discrepancies resolved through discussion with a third author (PE).

Statistical Analyses and Data Synthesis

Descriptive statistics were used to analyze the data for any substantial characteristics of the variables in the studies. The maximally adjusted odds ratios (ORs) with associated 95% confidence intervals (CIs) were generated from each study, and summary estimates were calculated using random-effects inverse-variance modeling. The estimated statistical heterogeneity was assessed with the I² statistic. Due to the insufficient (<10) number of studies, the analysis was unable to include meta-regression.

Ethical Considerations

This paper contains no primary data obtained directly from research participants. Data obtained from previously published resources have been acknowledged within references.

Results

**Figure 1:**
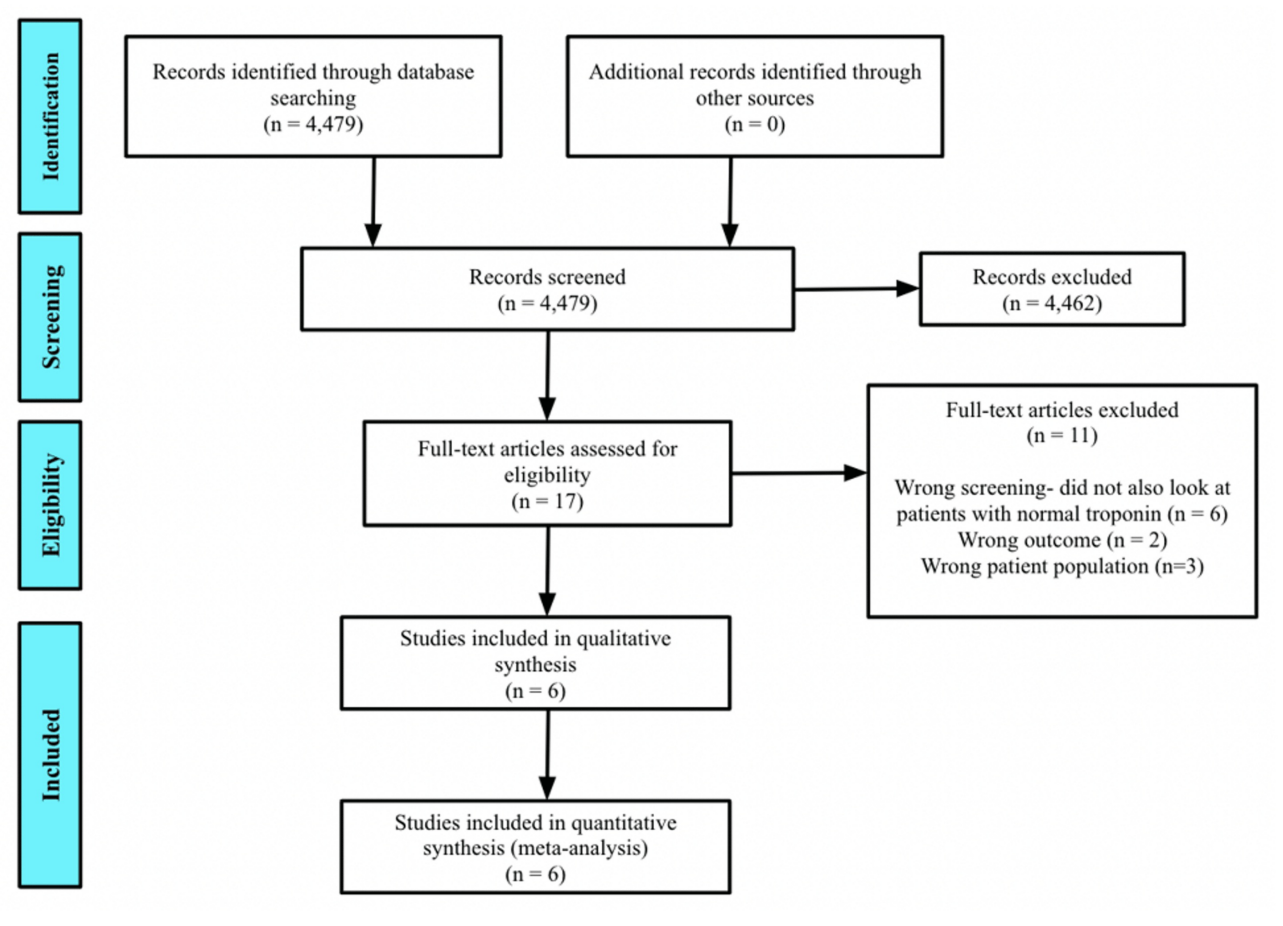
PRISMA study inclusion flow diagram PRISMA: Preferred Reporting Items for Systematic Reviews and Meta-Analyses

A total of 4,479 studies were identified. PubMed yielded 2,018 studies, and Cochrane yielded 2,451 studies. A total of 4,479 abstracts were reviewed, with 17 selected for full-text review (Figure 1). No additional papers were identified through bibliographic review.

Six studies, comprising a total of 3,144 patients, were selected for final analysis (Table 1). Some patients from the studies were isolated and taken out of the meta-analysis due to having known congenital cardiac conditions [27] and incomplete data [28]. All six studies were retrospective. Two studies focused solely on patients from the ED [27,29], while four studies examined pediatric patients throughout the hospital [28,30-32]. Four studies defined cardiac dysfunction by the patient's discharge diagnosis [27,29-31]. McGovern et al. defined it based on echocardiogram results [32], while Liesemer et al. defined it based on an abnormal electrocardiogram [28].

**Table 1:** Characteristics of the studies included in the analysis hs-cTnT, high-sensitivity cardiac troponin T; ED, emergency department; MRI, magnetic resonance imaging; ECHO, echocardiogram; ECG, electrocardiogram; TnT, cardiac troponin T; TnI, cardiac troponin I; Tn, troponin

Title	Design	Study Location	Sample Size	Age	Troponin Type	Troponin Threshold	Myocardial Injury	Outcomes Measured
Dionne et al. [30]	Retrospective Study	ED and inpatient	1,993	<21 years	TnT	≥0.1 ng/mL	Cardiac diagnosis at discharge	ECG, ECHO, cardiac MRI, or cardiac catheterization
Eisenberg et al. [31]	Retrospective Cohort Study	Anywhere in the hospital	221	<21 years	TnT	≥0.01 ng/mL	Cardiac diagnosis	Cardiac MRI, ECG, ECHO, pathology
McGovern et al. [32]	Retrospective Chart Review	Anywhere in the hospital	566	<18 years	TnI	≥0.05 μg/L	ECHO	Systolic or diastolic dysfunction on ECHO. Dysfunction was presumed to be absent if the patient had a normal initial cardiac assessment with or without ECHO or cardiology consultation and did not die or re-present with myocardial dysfunction.
Wang et al. [27]	Retrospective Cohort Study	ED	356	<18 years	hs-cTn	>99th percentile of the upper reference limit 15 ng/L for males and 10 ng/L for females	Discharge Diagnosis	ECG, pediatric cardiology consult, ECHO, cardiac MRI
Brancato et al. [29]	Retrospective Observational Study	ED	91	<19 years	Cardiac Tn (consists of 3 subunits: TnI, TnT, and troponin C)	0.03 ng/mL	Discharge Diagnosis	ECG, chest X-ray, ECHO, cardiac MRI, cardiac computed tomography
Liesemer et al. [28]	A Multicenter, Retrospective Review	ED, inpatient, or outpatient	3,497	<18 years	TnI	0.4 ng/mL	Abnormal ECG	ECG, ECHO, discharge diagnosis

The observed effect size estimates ranged from 1.80 to 5.38, with all estimates being positive. The estimated overall effect size for the combined study indicated a significant difference from zero (z = 3.298, p < .001) (Table 2).

**Table 2:** Wald test showing the overall effect size of the combined study

Statistic	Estimate	Standard Error	z	p	95% Confidence Interval	
					Lower	Upper
Intercept	0.563	0.563	5.853	< .001	2.194	4.403

The Q-test for heterogeneity indicated that the studies were statistically significantly heterogeneous (Q(5) = 39.034, p < .001, tau² = 1.430, I² = 87.313%) (Table 3).

**Table 3:** Residual heterogeneity estimates for FEM: Q-test for heterogeneity FEM, fixed effects model

Statistic	Estimate	95% Confidence Interval	
		Lower	Upper
τ^2^	1.430	0.342	11.278
τ	1.196	0.585	3.358
I^2 ^(%)	87.313	62.224	98.190
H^2^	7.882	2.647	55.261

**Figure 2:**
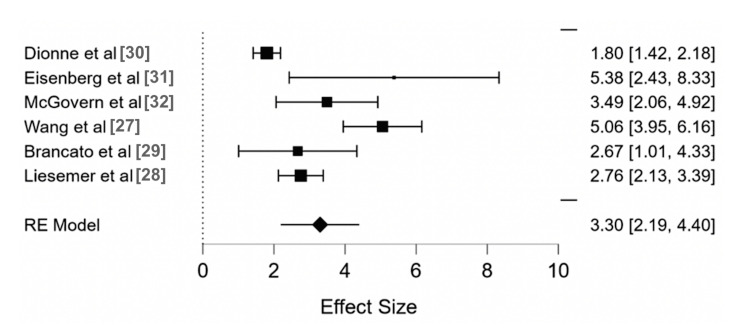
Forest diagram of the odds ratio between troponin and cardiac dysfunction among pediatric patients using the random effects restricted maximum likelihood method RE, random effects

The 95% prediction interval for the true outcomes was 2.194 and 4.403. The Random Effects Restricted Maximum Likelihood Method (REML) indicated a combined effect size of 3.30 (2.19, 4.40). Funnel plot analysis demonstrates some risk of publication bias (Figure 2).

**Figure 3:**
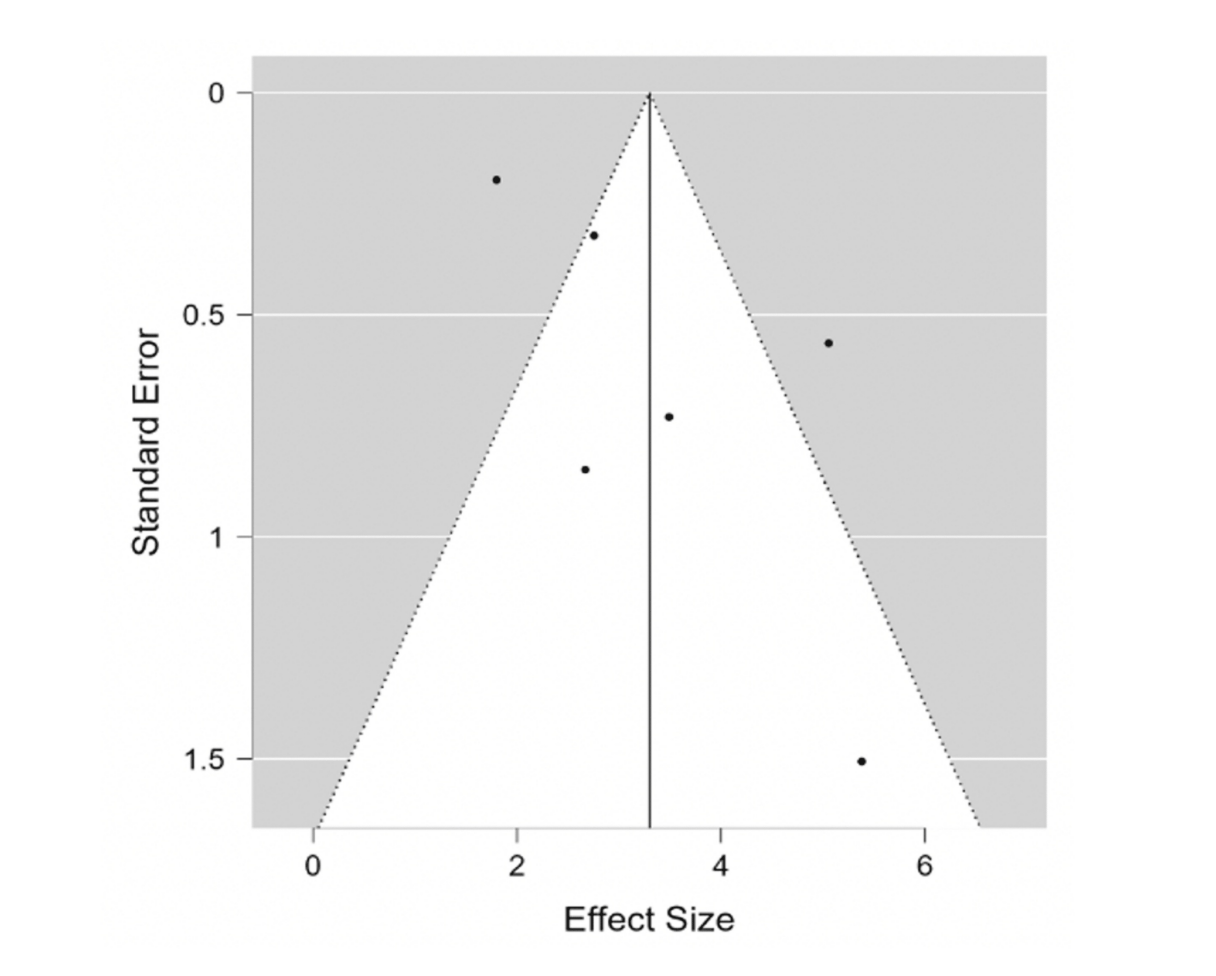
Funnel plot of the included studies

The rank correlation test for funnel plot asymmetry was not significant (τ = 0.333, p = 0.469), nor was the regression test for funnel plot asymmetry significant (z = 1.641, p = 0.101) (Figure 3).

Discussion

Key Findings

This meta-analysis investigated the association between troponin levels and myocardial dysfunction in pediatric patients presenting with chest pain. Our findings demonstrate a statistically significant correlation, with an OR of 3.30 (95% CI, 2.19-4.40), indicating that children with elevated troponin levels have a higher risk of myocardial dysfunction compared to those without elevated troponin levels. Despite the substantial heterogeneity among studies (I² = 87%), the results suggest that elevated troponin can be a useful prognostic biomarker for cardiac dysfunction in this population. However, there are limitations to using troponin as a screening tool, including the possibility of false positives due to non-cardiac conditions or physical activity-related injuries in children.

Implications

Previous studies have predominantly focused on the adult population, where troponin is a well-established marker for myocardial injury and prognosis in acute coronary syndrome [16,33]. In contrast, pediatric populations have been less studied, and the causes of chest pain in children are often non-cardiac [1,2,5,20]. This study helps bridge this gap by systematically reviewing and synthesizing existing evidence.

The findings of this meta-analysis suggest that elevated troponin levels should prompt further cardiac evaluation in pediatric patients. While elevated troponin alone should not be used as a definitive diagnostic tool, it can be an important component of a broader diagnostic workup. Clinicians should consider incorporating troponin measurements into their assessment protocols, especially when clinical suspicion of myocardial injury is high. However, caution is warranted when looking at troponin. Elevated troponin should be interpreted in the context of clinical findings and other diagnostic tests, such as echocardiography and electrocardiography.

Limitations

The primary limitation of this meta-analysis is the considerable heterogeneity among included studies. The heterogeneity observed may stem from differences in study design, troponin assay types, and thresholds used for defining troponin elevation, as well as variations in patient populations and settings. Although we used a random-effects model to account for this variability, the high I² statistic indicates that the results should be interpreted with caution. A further limitation related to heterogeneity is the small sample size, which limits further subgroup analysis or sensitivity analysis based on troponin subtype, threshold level, or care setting (ED vs. inpatient) to explore and potentially reduce the observed heterogeneity.

Additionally, the retrospective nature of all included studies may introduce bias, such as selection bias and information bias, which could affect the validity of the findings. The funnel plot analysis suggested a potential risk of publication bias.

Future Directions

Further high-quality prospective studies are needed to validate the findings of this meta-analysis. These studies should aim to include diverse populations across multiple sites and utilize consistent definitions and measurements of troponin elevation and myocardial dysfunction.

## Conclusions

The existing literature suggests that elevated troponin is associated with a higher risk of cardiac dysfunction. However, the data are limited by predominantly retrospective study designs, different outcomes examined, and a variance of troponin types used. Given the potential for identifying cardiac involvement and injury, allowing for early detection and intervention, and decreasing morbidity and mortality rates in children, troponin may be considered a useful diagnostic tool in the ED; however, further studies are needed.

## References

[1] Chen L, Duan H, Li X, Yang Z, Jiao M, Sun K, Jin M (2021). The causes of chest pain in children and the criteria for targeted myocardial enzyme testing in identifying the causes of chest pain in children. Front Cardiovasc Med.

[2] Drossner DM, Hirsh DA, Sturm JJ (2011). Cardiac disease in pediatric patients presenting to a pediatric ED with chest pain. Am J Emerg Med.

[3] Fogliazza F, Cifaldi M, Antoniol G, Canducci N, Esposito S (2024). Approaches to pediatric chest pain: a narrative review. J Clin Med.

[4] Zhang PI, Hsu CC, Kao Y (2020). Real-time AI prediction for major adverse cardiac events in emergency department patients with chest pain. Scand J Trauma Resusc Emerg Med.

[5] Chen L, Duan H, Li G, Li X (2022). The etiology of chest pain in children admitted to cardiology clinics and the use echocardiography to screen for cardiac chest pain in children. Front Pediatr.

[6] Lizano Santamaria RW, Morgan CT, Jeewa A, Dragulescu A (2023). Cardiac ischemia in pediatrics. Pediatr Rev.

[7] Alsabri M, Elshanbary AA, Nourelden AZ (2024). Chest pain in pediatric patients in the emergency department-presentation, risk factors and outcomes-a systematic review and meta-analysis. PLoS One.

[8] Lu JC, Bansal M, Behera SK (2017). Development of quality metrics for ambulatory pediatric cardiology: chest pain. Congenit Heart Dis.

[9] Nguyen T, Fundora MP, Welch E (2017). Application of the pediatric appropriate use criteria for chest pain. J Pediatr.

[10] Alnaim AA, AlGarni HW, Al Ghadeer HA (2023). Characteristics of chest pain among children presenting to the pediatric emergency department. J Med Life.

[11] Clerico A, Zaninotto M, Padoan A (2019). Evaluation of analytical performance of immunoassay methods for cTnI and cTnT: from theory to practice. Adv Clin Chem.

[12] Eerola A, Poutanen T, Savukoski T, Pettersson K, Sairanen H, Jokinen E, Pihkala J (2014). Cardiac troponin I, cardiac troponin-specific autoantibodies and natriuretic peptides in children with hypoplastic left heart syndrome. Interact Cardiovasc Thorac Surg.

[13] Yoldaş T, Örün UA (2019). What is the significance of elevated troponin I in children and adolescents? A diagnostic approach. Pediatr Cardiol.

[14] Neves AL, Henriques-Coelho T, Leite-Moreira A, Areias JC (2016). Cardiac injury biomarkers in paediatric age: are we there yet?. Heart Fail Rev.

[15] Bohn MK, Steele S, Hall A, Poonia J, Jung B, Adeli K (2021). Cardiac biomarkers in pediatrics: an undervalued resource. Clin Chem.

[16] Borg Caruana C, Jackson SM, Ngyuen Khuong J (2020). Systematic review and meta-analysis of postoperative troponin as a predictor of mortality and major adverse cardiac events after vascular surgery. J Vasc Surg.

[17] Costache AD, Leon-Constantin MM, Roca M (2022). Cardiac biomarkers in sports cardiology. J Cardiovasc Dev Dis.

[18] Bohn MK, Higgins V, Kavsak P, Hoffman B, Adeli K (2019). High-sensitivity generation 5 cardiac troponin T sex- and age-specific 99th percentiles in the CALIPER cohort of healthy children and adolescents. Clin Chem.

[19] Zrinski Topic R, Lenicek Krleza J (2025). Cardiac markers in pediatric laboratory medicine: critical review. Diagnostics (Basel).

[20] Guyther J, Cantwell L (2021). Big tests in little people. Emerg Med Clin North Am.

[21] Forero DA, Lopez-Leon S, González-Giraldo Y, Bagos PG (2019). Ten simple rules for carrying out and writing meta-analyses. PLoS Comput Biol.

[22] Stroup DF, Berlin JA, Morton SC (2000). Meta-analysis of observational studies in epidemiology: a proposal for reporting. JAMA.

[23] Blum RW, Hirsch D, Kastner TA (2002). A consensus statement on health care transitions for young adults with special health care needs. Pediatrics.

[24] Sawyer SM, McNeil R, Francis KL (2019). The age of paediatrics. Lancet Child Adolesc Health.

[25] Barak S, Rubino A, Grguric J, Ghenev E, Branski D, Olah E (2010). The future of primary paediatric care in Europe: reflections and Report of the EPA/UNEPSA Committee. Acta Paediatr.

[26] Jaffe AS, Ordonez-Llanos J (2013). High-sensitivity cardiac troponin: from theory to clinical practice. Rev Esp Cardiol (Engl Ed).

[27] Wang AP, Homme JL, Qureshi MY, Sandoval Y, Jaffe AS (2022). High-sensitivity troponin T testing for pediatric patients in the emergency department. Pediatr Cardiol.

[28] Liesemer K, Casper TC, Korgenski K, Menon SC (2012). Use and misuse of serum troponin assays in pediatric practice. Am J Cardiol.

[29] Brancato F, De Rosa G, Gambacorta A (2021). Role of troponin determination to diagnose chest pain in the pediatric emergency department. Pediatr Emerg Care.

[30] Dionne A, Kheir JN, Sleeper LA, Esch JJ, Breitbart RE (2020). Value of troponin testing for detection of heart disease in previously healthy children. J Am Heart Assoc.

[31] Eisenberg MA, Green-Hopkins I, Alexander ME, Chiang VW (2012). Cardiac troponin T as a screening test for myocarditis in children. Pediatr Emerg Care.

[32] McGovern E, Voss C, Hemphill NM, Sanatani S, Barakauskas V, Harris KC (2021). Evaluation of conventional troponin I testing for the detection of myocardial dysfunction in children. Paediatr Child Health.

[33] Nagarajan V, Hernandez AV, Tang WH (2012). Prognostic value of cardiac troponin in chronic stable heart failure: a systematic review. Heart.

